# Long-Term Efficacy of Bariatric Surgery Compared to Modern Medical Therapy in Type 2 Diabetes and Obesity: A Systematic Review

**DOI:** 10.7759/cureus.89409

**Published:** 2025-08-05

**Authors:** Ateeq Afzal, Jaya Ram Pandey, Tehreem Ashraf, Ali Jamal Ashraf Bagri, Mehak Kumari, Shivam Singla, Bhavna Singla, Nabila N Anika, Ahmad Irshad

**Affiliations:** 1 Pediatrics, District Civil Hospital, Hafizabad, PAK; 2 General Surgery, Nepalese Army Institute of Health Sciences, Kathmandu, NPL; 3 Internal Medicine, Islam Medical and Dental College, Sialkot, PAK; 4 Internal Medicine, Chandka Medical College, Larkana, PAK; 5 Internal Medicine, TidalHealth Peninsula Regional, Salisbury, USA; 6 Internal Medicine, Erie County Medical Center, Buffalo, USA; 7 General Surgery, Baylor College of Medicine, Houston, USA; 8 Medicine and Surgery, Holy Family Red Crescent Medical College Hospital, Dhaka, BGD; 9 Internal Medicine, Combined Military Hospital, Muzaffarabad, PAK

**Keywords:** bariatric surgery, glp-1 receptor agonists, glycemic control, medical therapy, metabolic surgery, obesity, randomized controlled trials, sglt2 inhibitors, type 2 diabetes mellitus, weight loss

## Abstract

This systematic review evaluates the comparative effectiveness of bariatric surgery versus medical therapy in managing obese patients with type 2 diabetes mellitus (T2DM). A decade-long literature search from January 2014 to January 2024 identified 10 randomized controlled trials (RCTs) involving diverse populations, interventions, and outcomes. The analysis demonstrates that bariatric procedures, such as Roux-en-Y gastric bypass (RYGB), sleeve gastrectomy (SG), and metabolic surgery, consistently outperform medical interventions, including GLP-1 receptor agonists, SGLT2 inhibitors, and intensive lifestyle modifications, in achieving superior glycemic control, weight reduction, and metabolic improvement. Surgical interventions also led to favorable outcomes in terms of quality of life, beta-cell function, and renal protection, particularly in high-risk groups such as adolescents and patients with diabetic kidney disease. The risk of bias assessment showed that most trials were of low risk or some concern, lending moderate to high confidence to the conclusions. These findings highlight the potential of metabolic surgery as a transformative strategy for durable diabetes remission in appropriately selected patients, supporting its integration into clinical guidelines alongside pharmacotherapy.

## Introduction and background

Obesity and type 2 diabetes mellitus (T2DM) are two of the most pressing public health challenges of the 21st century, with rapidly increasing global prevalence [1]. Obesity is a major risk factor for the development of insulin resistance, which plays a pivotal role in the pathogenesis of T2DM. As both conditions often coexist, their combined impact exacerbates the risks of cardiovascular disease, chronic kidney disease, and mortality, while posing a substantial economic burden on healthcare systems [2].

Standard management of T2DM in obese individuals primarily involves lifestyle modifications, dietary changes, physical activity, and pharmacological interventions, including insulin, metformin, glucagon-like peptide-1 (GLP-1) receptor agonists, and sodium-glucose co-transporter-2 (SGLT2) inhibitors [3]. Although these approaches help achieve glycemic targets, they rarely lead to long-term remission and often fail to address the underlying metabolic dysregulation and excess adiposity [4].

In contrast, bariatric or metabolic surgery, including procedures such as Roux-en-Y gastric bypass (RYGB) and sleeve gastrectomy (SG), has demonstrated substantial efficacy in improving glycemic control and, in many cases, achieving remission in patients with T2DM [5]. These surgical interventions are increasingly recognized for their metabolic benefits beyond weight loss, with growing evidence indicating reductions in cardiovascular risk, improvements in renal function, and enhanced health-related quality of life (HRQoL) [6]. However, the optimal selection criteria for surgical candidates, long-term safety, cost-effectiveness, and comparative efficacy versus pharmacological therapy remain subjects of ongoing investigation [7]. The objective of this systematic review is to evaluate and compare the clinical outcomes of bariatric surgery with those of evidence-based pharmacological treatment regimens, such as metformin, insulin, GLP-1 receptor agonists, and SGLT2 inhibitors, in adults with obesity and T2DM. Key outcomes assessed include glycemic control (e.g., HbA1c levels), diabetes remission, weight reduction, HRQoL, and the incidence of long-term complications.

## Review

Materials and methods

Study Design and Reporting Framework

This systematic review was designed in accordance with the Preferred Reporting Items for Systematic Reviews and Meta-Analyses (PRISMA) 2020 guidelines [8] to ensure methodological transparency and reproducibility. A structured PICO (Population, Intervention, Comparison, Outcome) framework [9] was utilized to guide the study focus. The population included obese patients with a confirmed diagnosis of T2DM. The interventions studied were bariatric surgical procedures, such as RYGB, SG, and transit bipartition. These were compared against pharmacological therapy and lifestyle interventions, including treatments with metformin, insulin, GLP-1 receptor agonists, SGLT2 inhibitors, and structured lifestyle modifications. Outcomes assessed included glycemic control (e.g., HbA1c), body weight, body mass index (BMI), insulin sensitivity (as inferred from changes in fasting plasma glucose, insulin requirements, and in select studies, molecular markers such as oxidative stress and beta-cell viability), kidney function (e.g., hyperfiltration, urinary albumin excretion), bone turnover markers (e.g., CTX, osteocalcin), and quality of life measures.

Search Strategy and Eligibility Criteria

A comprehensive literature search was conducted using PubMed, Embase, the Cochrane Central Register of Controlled Trials (CENTRAL), and Scopus to identify relevant randomized controlled trials (RCTs) published from January 2014 to February 2025. Although the initial search timeframe was up to January 2024, the database was updated during manuscript preparation to capture eligible trials published ahead of print or made available online in early 2025. The search strategy incorporated Medical Subject Headings (MeSH) and free-text terms combined with Boolean operators, including keywords such as "bariatric surgery", "metabolic surgery", "Roux-en-Y gastric bypass", "sleeve gastrectomy", "gastric bypass", "obesity", "overweight", "type 2 diabetes", "T2DM", "GLP-1 receptor agonist", "SGLT2 inhibitor", "metformin", and "randomized controlled trial". Only peer-reviewed, full-text articles published in English were included. Eligible studies enrolled adult or adolescent participants with obesity or overweight and a confirmed diagnosis of T2DM. Studies were required to involve clearly defined surgical or pharmacological interventions and report at least one relevant clinical outcome, such as glycemic control, weight change, or quality of life, with a minimum follow-up duration of six months.

Study Selection and Data Extraction

After removal of duplicates, two independent reviewers screened titles and abstracts for relevance. Full texts of potentially eligible studies were reviewed to determine final inclusion based on predefined criteria. Data were extracted independently using a standardized form, capturing details on study design, sample size, participant demographics, intervention and comparator specifics, follow-up duration, and key surgical and medical outcomes. Any disagreements were resolved through discussion and consensus.

Risk of Bias Assessment

The Cochrane Risk of Bias Tool (RoB 2) [10] for randomized trials was used to assess the methodological quality of the included studies. Domains assessed included the randomization process, deviations from intended interventions, missing outcome data, measurement of the outcome, and selection of the reported result. Most included studies were found to have a low risk of bias or some concerns, indicating generally reliable evidence quality.

Data Synthesis

Due to the clinical and methodological heterogeneity across studies, a narrative synthesis approach was employed. This allowed for a qualitative comparison of surgical and medical interventions across different populations, outcomes, and study durations without the need for quantitatively pooling data. The structured and comprehensive methodology ensures that the conclusions derived from this review are grounded in rigorously selected and critically appraised evidence.

Results

Study Selection Process

**Figure 1:**
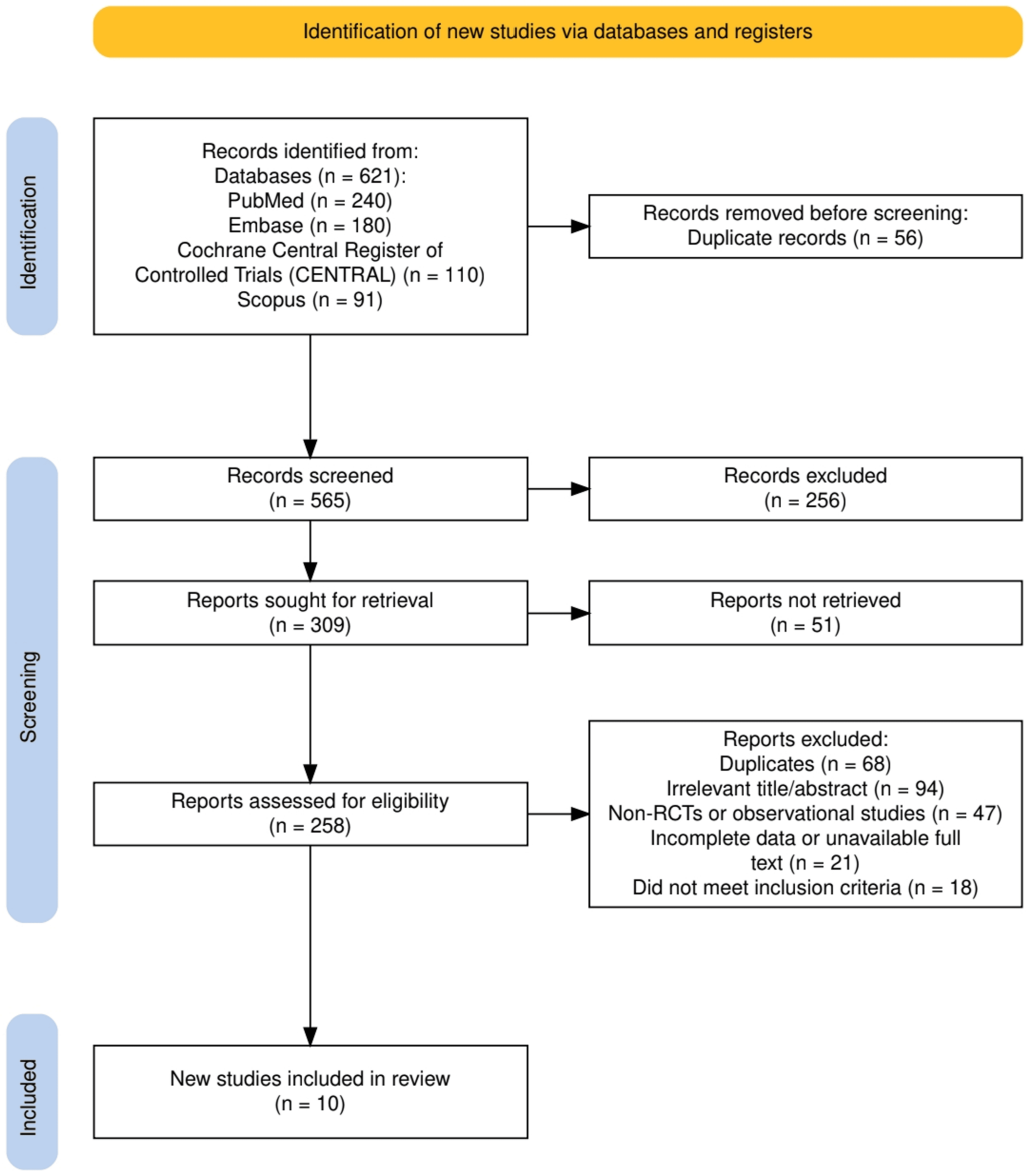
The PRISMA flowchart represents the study selection process. PRISMA: Preferred Reporting Items for Systematic Reviews and Meta-Analyses

As illustrated in Figure 1, a total of 621 records were identified through database searches: PubMed (n = 240), Embase (n = 180), Cochrane Central Register of Controlled Trials (CENTRAL) (n = 110), and Scopus (n = 91). After removing 56 duplicates, 565 records were screened. Of these, 256 were excluded based on title or abstract, and 51 full-text reports could not be retrieved. The remaining 258 reports were assessed for eligibility. Following further exclusions due to duplication (n = 68), irrelevance (n = 94), study design (n = 47), incomplete data (n = 21), and unmet inclusion criteria (n = 18), 10 RCTs were included in the final systematic review.

Characteristics of the Selected Studies

As summarized in Table 1, the 10 included RCTs involved both adult and adolescent populations with obesity and T2DM. Sample sizes ranged from 20 to 228 participants, with follow-up periods extending from six months to 12 years. Bariatric procedures evaluated included RYGB, SG, biliopancreatic diversion, and transit bipartition. These were compared against medical and lifestyle interventions comprising metformin, insulin, GLP-1 receptor agonists, SGLT2 inhibitors, or combinations thereof. Across trials, surgical groups consistently demonstrated greater improvements in glycemic control (HbA1c), diabetes remission, weight loss, renal outcomes, lipid profiles, and HRQoL compared to medical therapy alone. Several studies also highlighted specific advantages in adolescents, patients with early diabetic kidney disease, and those with moderate obesity.

**Table 1:** Summary of randomized controlled trials comparing bariatric surgery and medical therapy in T2DM and obesity. T2DM: Type 2 Diabetes Mellitus; BMI: Body Mass Index; RYGB: Roux-en-Y Gastric Bypass; SG: Sleeve Gastrectomy; GLP-1 RA: Glucagon-Like Peptide-1 Receptor Agonist; SGLT2i: Sodium-Glucose Co-Transporter-2 Inhibitor; FGF19: Fibroblast Growth Factor 19; GIP: Glucose-Dependent Insulinotropic Polypeptide; QoL: Quality of Life; FPG: Fasting Plasma Glucose; UAE: Urinary Albumin Excretion; DKD: Diabetic Kidney Disease; HRQoL: Health-Related Quality of Life; PTH: Parathyroid Hormone; CTX: C-Terminal Telopeptide; OC: Osteocalcin

Study (Author, Year)	Study Design	Sample Size (Surgery/Medical)	Population Characteristics (Age, BMI, T2DM Duration)	Intervention	Comparator	Duration of Follow-Up	Outcome
Schauer et al., 2017 [11]	Randomized Controlled Trial	96/54	Mean age: 49±8 yrs, BMI: 37±3.5, T2DM patients with BMI 27–43	Roux-en-Y gastric bypass or sleeve gastrectomy + intensive medical therapy	Intensive medical therapy alone	5 years	29% (gastric bypass) and 23% (sleeve gastrectomy) achieved HbA1c ≤6.0% vs 5% in medical therapy; greater reductions in weight, triglycerides, insulin use, and improved QoL in surgical groups
Mingrone et al., 2015 [12]	Randomized Controlled Trial	40/20	Age 30–60 yrs, BMI ≥35, T2DM ≥5 years	Roux-en-Y gastric bypass or biliopancreatic diversion	Conventional medical therapy	5 years	50% of surgical patients maintained diabetes remission vs. 0% in the medical group; better HbA1c control, lower cardiovascular risk, and fewer diabetes-related complications in the surgical group
Simonson et al., 2025 [13]	Randomized Controlled Trial	152/76	Mean age: 49.2±8.0 yrs, BMI: 36.3±3.4, HbA1c: 8.7±1.6%	Roux-en-Y gastric bypass, sleeve gastrectomy, or gastric band	Medical/lifestyle intervention	Up to 12 years	MBS improved the physical component of HRQoL, vitality, general health, and health utility more than MLI; BMI reduction correlated with better physical outcomes; mental health changes were minimal
Aminian et al., 2021 [14]	Randomized Controlled Trial	Subset of 104 from STAMPEDE trial	T2DM patients with obesity; baseline general health was assessed	Roux-en-Y gastric bypass or sleeve gastrectomy	Intensive medical therapy	5 years	Surgery improved physical functioning, energy, general health, and diabetes-related QoL more than IMT; no significant change in psychosocial outcomes across groups
Inge et al., 2018 [15]	Randomized Controlled Trial	30/63	Adolescents (mean age ~16 yrs), severely obese with T2DM	Bariatric surgery (Teen-LABS cohort)	Medical therapy with metformin, rosiglitazone, or lifestyle changes (TODAY cohort)	2 years	Surgery resulted in improved HbA1c (from 6.8% to 5.5%), 29% BMI reduction, and improved comorbidities; the medical group had worsening glycemia and weight gain
Bhandari et al., 2017 [16]	Clinical Trial	30/60 (30 GLP-1 + 30 SGLT2)	Indian patients with BMI 30–35, T2DM duration ~3 yrs, HbA1c >7.5%	Bariatric surgery	GLP-1 analogues and SGLT2 inhibitors	12 months	All groups showed HbA1c and FPG reduction; changes were significantly greater in the surgery group, along with better lipid profile improvement
Azevedo et al., 2018 [17]	Randomized Controlled Trial	10/10	Male adults ≤65 yrs, BMI 28–35, HbA1c >8%, T2DM	Sleeve gastrectomy with transit bipartition (SG + TB)	Standard medical therapy (SMT)	24 months	SG + TB significantly reduced HbA1c (to 5.5%), BMI, and need for antihypertensive/lipid meds; improved HDL, GLP1, FGF19, and GIP responses vs. the SMT group
Constantin et al., 2019 [18]	Randomized Clinical Trial	Not specified	Obese males with T2DM	Laparoscopic sleeve gastrectomy (LSG)	Conventional therapy	6 months	Sera from the LSG group improved beta cell viability and proliferation, reduced ROS and ER stress markers, and enhanced insulin expression; no such changes in the medical group
Bjornstad et al., 2020 [19]	Randomized Controlled Trial	30/63	Severely obese adolescents with T2DM; Teen-LABS: mean age 16.9 yrs, BMI 54.4; TODAY: mean age 15.3 yrs, BMI 40.5	Metabolic bariatric surgery	Medical therapy with metformin, rosiglitazone, or lifestyle intervention	5 years	The surgery group showed decreased rates of hyperfiltration (21% to 18%) and elevated UAE (27% to 5%), while the medical group saw marked increases in both parameters, indicating lower DKD risk with surgery
Crawford et al., 2018 [20]	Randomized Controlled Trial	70/25	Mean age ~48.5 yrs, BMI 36.5 ± 3.6, uncontrolled T2DM (HbA1c 9.3 ± 1.6%)	Roux-en-Y gastric bypass or sleeve gastrectomy	Intensive medical therapy	5 years	Bone turnover markers (CTX and OC) increased significantly in surgery groups vs. medical therapy; the greatest increase in the RYGB group; associated with increased PTH and weight loss effects

Quality Assessment

As outlined in Table 2, the risk of bias across the included studies was generally low or raised some concerns. Most trials demonstrated robust methodology with proper randomization and consistent outcome measurement. However, some studies showed concerns related to deviations from intended interventions, incomplete outcome data, or limitations in randomization procedures. A few trials were rated as low risk in all assessed domains, reinforcing the credibility of their results. Others, with multiple domains marked as "some concerns," warrant cautious interpretation when integrating their findings into clinical conclusions.

**Table 2:** Risk of bias assessment of the included trials using the Cochrane RoB 2.0 tool. RoB: Risk of Bias; RCT: Randomized Controlled Trial; T2DM: Type 2 Diabetes Mellitus; BMI: Body Mass Index

Study (Author, Year)	Randomization	Deviations From Intended Interventions	Missing Outcome Data	Measurement of Outcomes	Selective Reporting	Overall RoB
Schauer et al., 2017 [11]	Low	Low	Low	Low	Low	Low Risk
Mingrone et al., 2015 [12]	Low	Some Concerns	Low	Low	Low	Some Concerns
Simonson et al., 2025 [13]	Low	Low	Some Concerns	Low	Low	Some Concerns
Aminian et al., 2021 [14]	Low	Low	Low	Low	Low	Low Risk
Inge et al., 2018 [15]	Some Concerns	Some Concerns	Some Concerns	Low	Low	Some Concerns
Bhandari et al., 2017 [16]	Some Concerns	Some Concerns	Some Concerns	Low	Low	Some Concerns
Azevedo et al., 2018 [17]	Low	Low	Low	Low	Low	Low Risk
Constantin et al., 2019 [18]	Some Concerns	Some Concerns	Low	Low	Low	Some Concerns
Bjornstad et al., 2020 [19]	Some Concerns	Some Concerns	Some Concerns	Low	Low	Some Concerns
Crawford et al., 2018 [20]	Low	Low	Some Concerns	Low	Low	Some Concerns

Discussion

This systematic review synthesizes high-quality evidence from 10 RCTs comparing the long-term efficacy of bariatric surgery with modern medical therapy in managing T2DM among obese individuals. Across the trials, bariatric interventions, including RYGB, SG, and transit bipartition, consistently led to superior outcomes in glycemic control, weight reduction, and overall metabolic health. These benefits were sustained over extended follow-up periods, with some trials tracking outcomes for up to 12 years. In contrast, medical interventions, even those using newer agents such as GLP-1 receptor agonists and SGLT2 inhibitors, showed limited capacity to achieve comparable results in disease remission or metabolic normalization.

Glycemic Control and Diabetes Remission

Across nearly all included studies, bariatric surgery resulted in significantly greater and more sustained reductions in HbA1c levels than pharmacological therapy. Several trials reported diabetes remission, defined as achieving an HbA1c level of ≤6.0% without the use of anti-diabetic medications, in 23-50% of surgical patients, whereas medical therapy, including metformin and insulin-based regimens, rarely achieved remission rates beyond 5% [11,12]. In subgroup analyses, bariatric surgery consistently outperformed monotherapies such as metformin and combination regimens involving insulin or GLP-1 receptor agonists. For example, Bhandari et al. [16] directly compared surgery with both GLP-1 analogues and SGLT2 inhibitors, demonstrating superior glycemic control and lipid profile improvements in the surgical group. These findings align with results from landmark studies such as the STAMPEDE trial [14], where surgical groups maintained glycemic superiority even among patients with shorter T2DM duration and lower BMI. The metabolic benefits of surgery are multifactorial, involving not only calorie restriction and weight loss but also improved insulin sensitivity, preservation of beta-cell function, and enhanced gut hormone regulation (e.g., GLP-1, PYY).

Weight Reduction and BMI Normalization

Substantial weight loss was a consistent finding across surgical groups, with average reductions exceeding 25-30% of baseline body weight, far exceeding the 5-10% typically achieved with standard medical therapy or lifestyle changes [13,15]. While pharmacologic agents, such as GLP-1 receptor agonists, have shown promising weight loss results, as seen in the STEP trials, which reported weight reductions of over 10%, the magnitude and durability of weight loss were generally greater in the surgical arms of the RCTs included in this review. Notably, Bhandari et al. [16] found that bariatric surgery outperformed both GLP-1 and SGLT2-based therapies in terms of body weight reduction at 12 months. In adolescents, the benefit was even more pronounced; Inge et al. [15] and Bjornstad et al. [19] observed that early metabolic surgery resulted in substantial BMI reductions, improved comorbidities, and reduced progression to severe metabolic dysfunction. These findings suggest that while newer pharmacotherapies are advancing, bariatric surgery remains the most effective intervention for achieving substantial and sustained weight loss in appropriate patients.

Renal and Cardiovascular Outcomes

A critical finding across several studies is the renal protective effect of bariatric surgery. For instance, Bjornstad et al. reported a marked reduction in hyperfiltration and urinary albumin excretion in adolescents undergoing metabolic surgery, indicating delayed onset or progression of diabetic kidney disease (DKD) [19]. Similarly, Mingrone et al. [12] and Aminian et al. [14] found that surgical groups had improved lipid profiles, lower blood pressure, and better control of albuminuria, which collectively reduce cardiovascular morbidity. These effects may stem from weight-independent pathways such as reduced inflammation, improved endothelial function, and neurohormonal changes.

Quality of Life and Functional Status

Several studies evaluated HRQoL using validated instruments. Across these, surgical groups reported higher scores in domains related to physical functioning, vitality, general health perception, and energy levels. Simonson et al. [13] and Aminian et al. [14] specifically linked BMI reduction with enhanced physical components of QoL. Interestingly, changes in mental health parameters were less consistent, possibly reflecting persistent psychosocial challenges, highlighting the need for integrated mental health support in postoperative care.

Comparative Effectiveness of Pharmacologic Therapy

While GLP-1 receptor agonists and SGLT2 inhibitors have demonstrated efficacy in glucose lowering and weight reduction, their effects plateau over time, and they rarely achieve diabetes remission. Bhandari et al. found significant reductions in HbA1c and fasting plasma glucose in medical groups receiving modern agents, but the magnitude of improvement was significantly greater in the surgical group [16]. Moreover, the surgical interventions often led to the discontinuation of insulin or oral hypoglycemics, a crucial benefit in reducing medication burden and associated side effects.

Mechanistic Insights: Why Surgery Works Better

The superiority of bariatric surgery stems from more than caloric restriction. Post-surgical patients exhibit elevated secretion of GLP-1, peptide YY (PYY), and fibroblast growth factor 19 (FGF19), which collectively improve glucose homeostasis and satiety. Changes in bile acid metabolism, gut microbiota, and insulin sensitivity further contribute to sustained glycemic control [21-23]. These physiological changes are not replicated by pharmacological agents alone. Importantly, Constantin et al. demonstrated that post-surgical serum enhances beta-cell viability and reduces oxidative stress, highlighting the deep metabolic reprogramming induced by surgery [18].

Subgroup Insights: Adolescents, Asians, and High-Risk Populations

Adolescents and South Asian populations appear to benefit substantially from early surgical intervention. Given their predisposition to visceral adiposity and early-onset T2DM, traditional therapies are often insufficient. Studies like those by Inge et al. [15] and Bhandari et al. [16] suggest that timely bariatric surgery in these groups may delay disease progression, prevent complications, and reduce lifetime medication needs. Additionally, patients with DKD, poor glycemic control, or high BMI despite therapy represent strong candidates for surgical consideration.

Clinical Implications and Future Guidelines

Our findings reinforce the need for updated clinical guidelines that position bariatric/metabolic surgery not as a last resort, but as a first-line intervention in appropriate patients. The consistent superiority of surgery in achieving remission, improving renal and cardiovascular health, and enhancing QoL calls for earlier surgical referral, especially in patients with poor response to medical therapy. These insights are aligned with emerging international recommendations, including those from the American Diabetes Association and the International Federation for the Surgery of Obesity.

Limitations and Research Gaps

Despite compelling evidence, several limitations must be acknowledged. Some of the included trials had relatively small sample sizes, limited follow-up durations, or lacked blinding, which may introduce performance and detection biases. Additionally, the majority of studies were conducted in high-income countries, leading to limited generalizability to low-resource settings where access to bariatric surgery is often constrained. There is also a notable scarcity of head-to-head comparisons between surgical interventions and newer pharmacologic regimens, such as the combination of GLP-1 receptor agonists and SGLT2 inhibitors. Future research should aim to address these gaps by conducting cost-effectiveness analyses across varied healthcare systems, investigating the impact of bariatric surgery on mental health and bone metabolism, evaluating the long-term (beyond 10 years) durability of diabetes remission and complication rates, and expanding clinical trials to include ethnically diverse and underserved populations.

## Conclusions

This systematic review provides robust evidence supporting the superior efficacy of bariatric surgery compared to pharmacological therapy in the management of T2DM among individuals with obesity. Surgical interventions consistently demonstrated sustained improvements in glycemic control, greater weight loss, and beneficial effects on renal function, cardiovascular risk factors, and quality of life. The consistency of these outcomes across multiple RCTs and diverse patient populations underscores their clinical relevance. While further research is warranted to optimize patient selection and assess long-term safety, current evidence supports the integration of metabolic surgery as a viable and effective treatment option for appropriately selected patients with T2DM and obesity. These findings highlight the potential role of bariatric surgery as more than a last-resort intervention but rather as a central component of comprehensive diabetes care when lifestyle and pharmacologic measures alone are insufficient.
